# The impact of child safety promotion on different social strata in a WHO Safe Community

**DOI:** 10.5249/jivr.v4i1.83

**Published:** 2012-01

**Authors:** Kent Lindqvist, Koustuv Dalal

**Affiliations:** ^*a*^Department of Medical and Health Sciences, Division of Social Medicine and Public Health Sciences, Linköping University, 581 83 Linköping, Sweden.

## Abstract

**Background:**

The objective of the current study was to evaluate outcomes of a program to prevent severe and less severe unintentional child injuries among the different social strata under WHO Safe Community program. Specifically, the aim was to study effectiveness of Safe Community program for reducing child injury.

**Methods:**

A quasi-experimental design was used, with pre- and post-implementation registrations covering the children (0 -15 years) in the program implementation area (population 41,000) and in a neighboring control municipality (population 26,000) in Östergötland County, Sweden.

**Results:**

Boys from not vocationally active households displayed the highest pre-intervention injury rate in both the control and intervention areas. Also in households in which the vocationally significant member was employed, boys showed higher injury rates than girls. Households in which the vocationally significant member was self-employed, girls exhibited higher injury rates than boys in the intervention area. After 6 years of program activity, the injury rates for boys and girls in employed category and injury rates for girls in self-employed category displayed a decreasing trend in the intervention area. However, in the control area injury rate decreased only for boys of employed families.

**Conclusions:**

The study indicated that almost no changes in injury rates in the control area suggested that the reduction of child injuries in the intervention area between 1983 and 1989 was likely to be attributable to the safety promotion program. Therefore, the current study indicates that Safe Community program seems to be successful for reducing child injuries.

## Introduction

Worldwide unintentional injuries remain a significant health problem for children, despite several decades of concerted efforts.^1^Among the children (0-15 years), most fatal injuries occur at home. Studies of child injury by severity suggest that the socioeconomic determinants of more severe injuries differ from those of less severe injuries.^1,2^However, less we know about the child injury prevention programs especially in relation to socioeconomic status of the children’s families.

Community based programs to prevent common non-fatal injuries have been effectively implemented as complements to various national safety programs.^3-7^The current study presents an outcome evaluation on different social strata of a program to prevent severe and less severe unintentional child injuries. The program was developed following the World Health Organization (WHO) Safe Community program (more details at http://www.phs.ki.se/csp/). Using a quasi-experimental design to compare intervention and control communities, the study investigated changes in the all-cause injury risk after program implementation. In addition, changes in the distribution of injury severity and injury event contexts in the intervention community were examined.^1^An assessment of the general structure and process of the program has previously been reported.^8^In Sweden, the positioning of the local government in the program structure appears to be the most important factor determining program effectiveness.

Recently WHO has published the world report on child injury and provided a call for drastic actions for childhood injury prevention.^9^WHO Safe Communities program has been operating for the last two decades to prevent injuries and promote safety. Earlier study indicated that, the relative risk for child injury has decreased significantly in a WHO Safe Community in Sweden without focusing socioeconomic determinants.^1^ Injuries especially of children have been reported to be more common in households with poorer social strata.^10,11^ Vulnerable populations living in poor social strata are disproportionately at a risk of injury.^12-15^However, to the best of authors knowledge, few studies to date have investigated the impact of child safety promotion programs on boys and girls from different social strata.^4^The current study addresses this gap in knowledge using WHO Safe Community program in Sweden.

The objective of the current study was to investigate differences in the distribution of the child injury rate reduction among the different social strata in the catchment area. Specifically, the aim was to study, using a quasi-experimental design,^16^rates of child injury treated by healthcare organizations among members of households at different levels of labour market integration before and after program implementation.

## Methods

The Motala community is one of the original reference sites for the World Health Organization (WHO) Safe Community accreditation criteria. The Safe Community concept was developed in Sweden in conjunction with the WHO, based on findings from local Swedish injury prevention programs in the 1970s and 1980s. Scandinavian countries were among the first to implement the Safe Community model in the late 1980s and early 1990s.^17^The model emphasizes community participation and multidisciplinary collaboration, recognizing that those most able to solve local injury problems are those people who live in that particular community.^7^

**Study design**

A quasi-experimental design was used, with pre- and post-implementation registrations covering the total populations 0-15 years of age in the program implementation area (Motala) and in a neighboring control municipality (Mjölby) in Östergötland County. The pre-implementation study period covered 52 weeks from 1 October 1983 to 30 September 1984. The post-implementation period covered 52 weeks from 1 January 1989 to 31 December 1989. Changes in the morbidity rates following the intervention were studied using prospective registration of all acute care episodes during the study period. The intervention area had four health care centers and a county annex hospital with a casualty department, while the control area shared the annex hospital and had two health care centers, one with an emergency unit.

**Implementation of the Motala program**

In 1985, the Health Services Board of the County Council and the Municipal Government Board agreed to share responsibility for a local injury prevention program and a self regulating Child Safety Council (CSC). CSC members included politicians, county officials whose departments were responsible for the care and welfare of children, and representatives of non-governmental organizations. In 1987, the CSC used its influence within the local social network to establish an organization for the regular implementation of safety measures. All injuries treated at health care units were reported to the program. The registration procedure was based on earlier experience in Sweden.^18^For all injured children treated at the emergency room at the local hospital, a form was filled in by staff with the time of contact and standard personal data. Statistical analyses identified high risk age groups, the most common injury environments, and the most common types.

The CSC cooperated with local mass media in the intervention area to provide regular information about injury prevention.

To reach preschool children, nurses in the intervention area were trained and asked to provide age adjusted safety information to parents at compulsory annual health visits. Follow up interviews with parents who had visited childcare nurses showed that almost all families had received the safety information. However, despite receiving the information, only a minority were aware of the major hazards. Therefore, a video demonstrating safety modifications in the home was distributed to all parents with children younger than 6 years of age as part of a behavioral safety education and information program directed at falls in the home. In addition, safety products and examples of modifications of risk environments were displayed at public places. Indoor environments at all daycare centers were also evaluated, but required only minor modifications. Regular safety rounds were introduced for safety maintenance at the daycare centers as well as at playgrounds and other public facilities frequented by preschool children. 

To target schoolchildren, indoor environments at schools and sports facilities were evaluated, and regular safety rounds for maintenance were also introduced. Furthermore, all physical education teachers in the intervention area participated in an injury prevention course focusing on high risk groups of children. This course was intended to contribute to meeting the goal that every child performing physical exercise would have the basic skills for the activity and be informed about rules and injury risks. Local sports clubs were also asked to contribute to the injury prevention program. For the most popular team sport, soccer, workshops for coaches and referees were used to discourage foul play. For the most popular individual sport, horseback riding, an attempt was made to support the supervision of novices, including new rules requiring supervision of young riders during all interaction with horses. 

Both structural and educational measures were taken to improve traffic safety. A "Safe way to school" program was implemented at every primary school in cooperation with the municipality's planning department. The program included a "Cut your garden hedge" initiative to increase driveway visibility in residential areas. In addition, voluntary organizations and the police provided traffic education programs aimed at primary and lower secondary school students. A one hour traffic lesson was scheduled each week for all fourth graders. Last, a safe cycling program was initiated to subsidize the price of cycle helmets and to promote helmet use. Children were also offered courses to "shape up your bike" to reduce risks of equipment failure. 

**Classification of data**

The Swedish Socio-economic Index (SEI) was used to classify the individuals in the study areas. The SEI was used since the early 1980s to represent social status in most national databases and statistics.^19^The SEI defines social status primarily as being based on occupation. Children and young people are categorized to the SEI group to which their parents' household belongs.

SEI data for all individuals in the intervention and control areas were collected from Statistics Sweden (http://www.scb.se). For the pre-implementation measurement, SEI data originated in the Census survey conducted in 1985. Corresponding data for the post-implementation measurement originated in the 1990 Census survey.

Considering that the WHO Safe Community model relies strongly on the existing civic social network, and that occupation is an important determinant for these networks, the detailed SEI categories were used for coding individuals into three secondary categories based on the relation that the household had to the labour market: (1) households in which the vocationally significant member was employed, i.e. the person in the household with the highest wage earnings; (2) households in which the vocationally significant member was an entrepreneur or self-employed; and (3) households in which the adults were not vocationally active.

**Community characteristics**

Motala is situated in the western part of the county of Östergötland. The population was approximately 41,000 during the study period (82% living in the central and residential areas and the 18% living in surrounding rural areas). Seventy seven percent were gainfully employed in the field of manufacturing, trade and public administration. Mjölby, control municipality area, (population 26,000), was selected on the basis of socio-economic and demographic similarities to Motala and obviously due to availability of injury data. The city of Mjölby is situated 30 km south of Motala in the same county in the south-eastern part of Sweden.

**Data collection**

All children and adolescents under 16 years of age arriving at any health care unit located in the intervention and control areas during the study periods were included in to the current study. The nature and event context of injuries was classified using the International Classification of Diseases, eighth revision,^20^and the abbreviated injury scale (AIS) was used to measure injury severity.^21^Based on information from medical records two specially trained nurses classified injuries after the care episode. The attending physician was asked to verify, whenever necessary the accuracy of the classification. However, due to a lack of resources data on injury severity and event context were not collected from the control area.^1^

To estimate the quality of the specific injury registration procedure, secondary sampling of all acute health care attendances in the intervention area was undertaken during the third week of the pre-implementation registration period and in both the intervention and control areas during the third week of the post-implementation registration period. University hospital emergency department records from September 1984 were also additionally analyzed for any systematic differences between persons from the intervention and control areas receiving care outside the care units providing data for this evaluation.

**Statistical methods**

Injury rates, expressed as per 100 person-years, were calculated by community (intervention and control municipality) for each study period (1983/1984 and 1989), by socio-economic group according to labour market: employed, self-employed and not vocationally active; and by gender, as well as for girls and boys together.^22^Ninety-five percent confidence intervals (CI) were employed for injury rates. To avoid double registration of the same injury, only the first episode of injury during each registration period was included in the calculations. However, if the child had any new other injury during the registration period - that was registered in the current study. The differences in injury rates between 1989 and 1983/1984 were computed for both areas with 95% CI. A P-value <0.05 was employed to test the level of statistical significance. Similarly, differences in changes of injury rate between the intervention and control areas were computed using the following expression:

Difference in changes of injury rate = [Post-intervention injury rate in intervention area – Pre-intervention injury rate in intervention area] – [Post intervention injury rate in control area – Pre-intervention injury rate in control area]

All computations were performed using SPSS statistical software (PASW Statistics, Version 18).

## Results

Less than 1% of the eligible patients could not be identified in the medical record archives for secondary data analyses. During 1983–84, child all-cause injury rates were 172 per 1000 population years in the intervention area and 124 per 1000 population years in the control area. This difference is due, in part, to a lower proportion of injured residents from the intervention area than in the control area seeking emergency care at the university hospital. Only 3% of residents from the intervention area were taken directly to the university hospital for care, compared with 12% from the control area.

The age and gender mix in both areas were close to the national average and stable over the registration periods. Members of households in which the vocationally significant member was employed constituted the largest share of the population < 16 years of age in both the intervention (84%) and control (82%) areas. The members of self-employed households represented 8% and 11%, respectively (Table 1).Members of households classified as not vocationally active constituted 8% and 7% respectively. The income levels in both areas were at 93% of the national average and remained stable between the registration periods. Between 49% and 51% of the total population in the intervention and control areas were gainfully employed during the registration periods. During both periods, the share of the population with more than compulsory school education was about 5% below the national average in both areas. Similarly, the share of urban residents remained between 79% and 82% in both areas. The distribution of employers was comparable between the areas and registration periods, the share employed by manufacturing industries (31–34%) was higher than the national average (21–20%).

**Table:** Table 1:**Populations 0 -15 years of age in the intervention and control areas displayed by sex and household relation to labour market employment**

Household	Intervention areas						Control areas					
	Boys		Girls		Total		Boys		Girls		Total	
	1983-84	1989	1983-84	1989	1983-84	1989	1983-84	1989	1983-84	1989	1983-84	1989
Employed	3431	3508	3447	3453	6878	6961	2225	2156	2138	2069	4363	4225
Self-employed	448	402	340	280	788	682	315	296	306	280	621	576
Not vocationally active	304	371	230	316	534	687	188	179	168	202	356	381
Total	4183	4281	4017	4049	8200	8330	2728	2631	2612	2551	5340	5182

Boys from not vocationally active households displayed the highest pre-intervention injury rate in both the control and intervention areas(Table 2). Also in households in which the vocationally significant member was employed, boys showed higher injury rates than girls. In the households where the vocationally significant members were self-employed, girls exhibited higher injury rates than boys in the intervention area.

**Table:** Table 2:**Rate per 100 person-years (95% confidence interval) of individuals 0 -15 years of age injured in 1983/1984 in intervention and control areas, displayed by sex and household relation to labour market employment.**

	Employed			Self-employed			Not vocationally active		
	Boys	Girls	Total	Boys	Girls	Total	Boys	Girls	Total
Intervention area	18.0(16.7, 19.2)	12.3(11.2, 13.4)	15.1(14.3, 16.0)	11.8(8.8, 14.8)	16.5(12.5, 20.4)	13.8(11.4, 16.2)	22.4(17.7, 27.1)	18.3(13.3, 23.3)	20.6(17.2, 24.0)
Control area	13.2(11.8, 14.6)	9.4(8.2, 10.7)	11.3(10.4, 12.3)	12.4(8.7, 16.0)	11.1(7.6, 14.6)	11.8(9.2, 14.3)	14.9(9.8, 20.0)	7.7(3.7, 11.8)	11.5(8.2, 14.8)

After 6 years of program activity, the injury rates for boys and girls in employed and for girls in self-employed categories displayed a decreasing trend in the intervention area(Table 3). However, in the control area injury rate decreased only for boys of employed families. Changes in injury rates in the control area were not statistically significant in other social strata.

**Table:** Table 3:**Rate per 100 person-years (95% confidence interval) of individuals 0 -15 years of age injured in 1989 and change in rates between 1989 and 1983/1984 (95% confidence interval) in intervention and control areas, displayed by sex and household relation to labour market employment.**

	Employed			Self-employed			Not vocationally active		
	Boys	Girls	Total	Boys	Girls	Total	Boys	Girls	Total
Intervention area	12.8(11.7, 13.9)	9.4(8.4, 10.4)	11.1(10.4, 11.8)	9.5(6.6, 12.3)	9.6(6.2, 13.1)	9.5(7.3, 11.5)	17.0(13.2, 20.8)	12.3(8.7, 16.0)	14.8(12.2, 17.5)
Change 1989–1983	-5.2(- 6.9, -3.5)	-2.9(-4.4, -1.5)	-4.0(-5.2, -2.9)	-2.4(-6.5, 1.8)	-6.8(-12.1, -1.6)	-4.3(-7.6, -1.0)	-5.4(-11.4, 0.7)	-5.9(-12.1, 0.3)	-5.8(-10.1, -1.4)
P value	0.000	0.000	0.000	0.263	0.013	0.011	0.078	0.055	0.008
Control area	11.1(9.8, 12.5)	8.7(7.4, 9.9)	9.9(9.0, 10.8)	12.8(9.0, 16.6)	12.5(8.6, 16.4)	12.7(10.0, 15.4)	12.8(7.9, 17.8)	10.9(6.6, 15.2)	11.8(8.6, 15.1)
Change 1989–1983	-2.0(-4.0, -0.1)	-0.8(-2.5,0.9)	-1.4(-2.7, -0.1)	0.5(-4.8, 5.7)	1.4(-3.8, 6.6)	0.9(-2.8, 4.6)	-2.0(-9.1, 5.0)	3.2(-2.7, 9.1)	0.3(-4.3, 4.9)
P value	0.039	0.368	0.032	0.865	0.602	0.628	0.571	0.302	0.901

Non-vocationally active households had the highest incidence of injury in the intervention area, and boys sustained injuries more frequently than girls in employed and non-vocational social status groups in both study areas.

## Discussion

The current study indicates that Safe Community program seems to be successful for reducing child injuries. The study analyzed the WHO Safe Community program for safety promotion with regard to associations between pre- and post-intervention injury rates among boys and girls, and socio-economic status, as defined by the employment category of the household's significant member. The study indicated that almost no changes in injury rates in the control area suggested that the reduction of child injuries in the intervention area between 1983 and 1989 was likely to be attributable to the safety promotion program. 

The socially disadvantaged children as indicated by the SEI categories were at the highest pre-intervention injury risk, indicating that lower socio-economic status is an important risk factor for injury; this is consistent with previous research.^15^The present study design did not allow for an investigation into the causes of these differences, although a possible explanation could be more prevalent use of unsafe domestic products and less attention/supervision of the guardians in deprived households. Another finding that requires further study is that girls in the self-employed category displayed higher injury rates than boys.

Considering the program's aim of equality in safety issues between social groupings, the program was only partially successful in that it reduced the injury rate in employed households but it did not influence the injury rate of the self-employed households for boys and not vocationally active households. This finding indicates that the program's approach of combining a population-based, community-wide strategy with more targeted interventions to groups at increased risk, i.e. children/teenagers, is not so much successful in reducing health inequalities. It has been suggested that a ‘pure’ population strategy is only appropriate when risk is widely diffused through the whole population.^16^Earlier studies have demonstrated that injuries in childhood are related both to poverty at the household level and to living in a deprived neighborhood, and that these influences are independent.^23^This evidence suggests a parallel use of community-wide efforts and more targeted area-based interventions in order to reduce child injuries. Another explanation could be substance abuse among adult members of non-vocationally active households, leading to accidents and/or neglect in child minding. While substance abuse has been associated with the occurrence of injury, the association has not been characterized by type of substance and injury type. However, a recent study has reported that alcohol and cocaine use is independently associated with violence-related injuries, whereas opiate use is independently associated with non-violent injuries and burns.^24^Screening for substance abuse was not included in the present study, which warrants to be addressed in future studies.^25^

The current study is from a medium size community in Sweden. As the socio-cultural characters vary over the areas, the current findings might suffer from making a general conclusion for Nordic countries. Therefore further evaluations are warranted in other WHO Safe Communities in other low-, medium- and high-income countries. For individuals injured more than once, only the first episode during each registration period was included in the current study. Repetitive injuries of the same nature of the same child warrant further studies. In future, similar studies are warranted using severity of the injuries. The study has used data from 1983-1989 to measure the changes of childhood injuries according to social status. Though the data seemed to be old but according to intention of the study it should not create any problem in connection to reality. The current context of the study can demand similar studies using recent data.

In conclusion, the Safe Community program seemed to be effective in that it reduced the childhood injury rates in the intervention areas. However, households with employed and self-employed (with exception for boys) revealed statistically significant social stratum for effective child injury intervention. The findings do seem to suggest that additional research on the issue of parental sex and gender role as it relates to employment status or self-employment could be an interesting area for further analysis and research. Further research on evaluation of the WHO Safe Community programs in association with social strata and child injury intervention is also warranted from different countries.
